# Supplementary material for: Pulsed Dipolar EPR for Self-Limited Complexes of Oligonucleotides Studies
Source: Biomolecules. 2024 Jul 23;14(8):887. doi: 10.3390/biom14080887 (PMC11351890; doi:10.3390/biom14080887)
Supplement: Supplementary file 1 [file biomolecules-14-00887-s001.zip › biomolecules-3061331-supplementary.pdf]

SUPPORTING INFORMATION

Pulsed Dipolar EPR for Self-limited Complexes of Oligonucleotides Studies

Alexey S. Chubarov [1,*], Burkhard Endeward [2], Maria A. Kanarskaya [1], Yuliya F. Polienko [3], Thomas F. Prisner [2] and Alexander A. Lomzov [1,*]

1 Institute of Chemical Biology and Fundamental Medicine SB RAS, 630090 Novosibirsk, Russia; makanarskaya@gmail.com (M.K.)

2 Institute of Physical and Theoretical Chemistry, Goethe University Frankfurt Max-von-Laue-Str. 7, 60438 Frankfurt am Main, Germany; B.Endeward@chemie.uni-frankfurt.de (B.E.), prisner@chemie.uni-frankfurt.de (T.P.)

3 N.N. Vorozhtsov Institute of Organic Chemistry SB RAS, 630090 Novosibirsk, Russia; polienko@nioch.nsc.ru (Y.P.)

* Correspondence: chubarovalesha@mail.ru and chubarov@niboch.nsc.ru (A.C.); lomzov@niboch.nsc.ru (A.L.);
Tel.: +7-913-763-1420 (A.C.); +7-913-009-2889 (A.L.)

Table of contents

Continuous wave EPR 2

Gel shift assay analysis 2

Thermal denaturation analysis (Melting analysis) 3

Data processing of PELDOR data 4

Molecular dynamics simulation and analysis 6

Comparison of PELDOR and molecular dynamics distance distribution for T_7_T_3*, T_{10}_T_3*, T_{25}_T_3*, and T_3*_T_{25} complexes 13

Continuous wave EPR

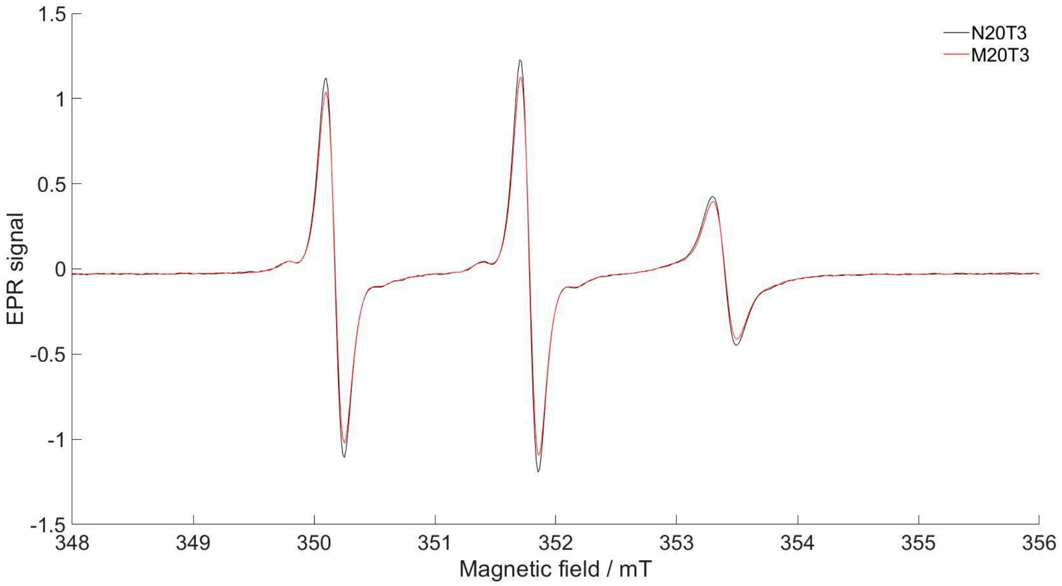

Figure S1. X-band CW EPR spectra of spin-labeled oligonucleotide M20T_3* and N20T_3* samples were measured at room temperature (For parameters, see section 2.6 of the paper).

Gel shift assay analysis

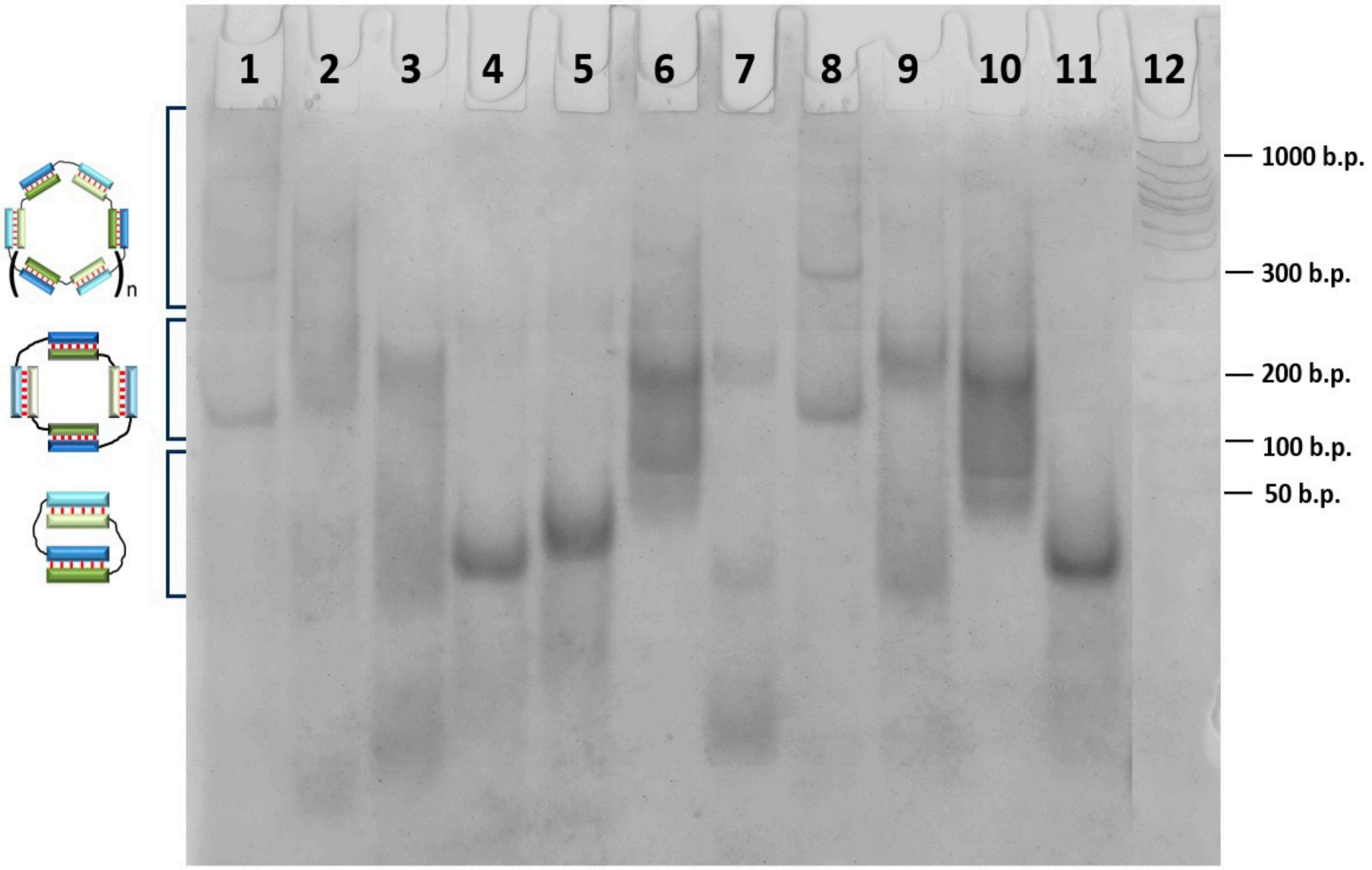

Figure S2. Gel shift assay of oligonucleotide complexes in a ratio of 1:1. Lane 1 T_0_T_3*, lane 2 T_1_T_3*, lane 3 T_3_T_3*, lane 4 T_7_T_3*, lane 5 T_{10}_T_3*, lane 6 T_{25}_T_3*, lane 7 T_3*_T_3*, lane 8 T_3*_T_0, lane 9 T_3*_T_3, lane 10 T_3*_T_{25}, lane 11 T_7_T_3 control, lane 12 ladder (50-1000 bp). A DNA Ladder of 50–1000 bp (SibEnzyme, Russia) was used to evaluate the mobility of complexes. Stains-all (Sigma, USA) was used to stain the gel.

Thermal denaturation analysis (Melting analysis)

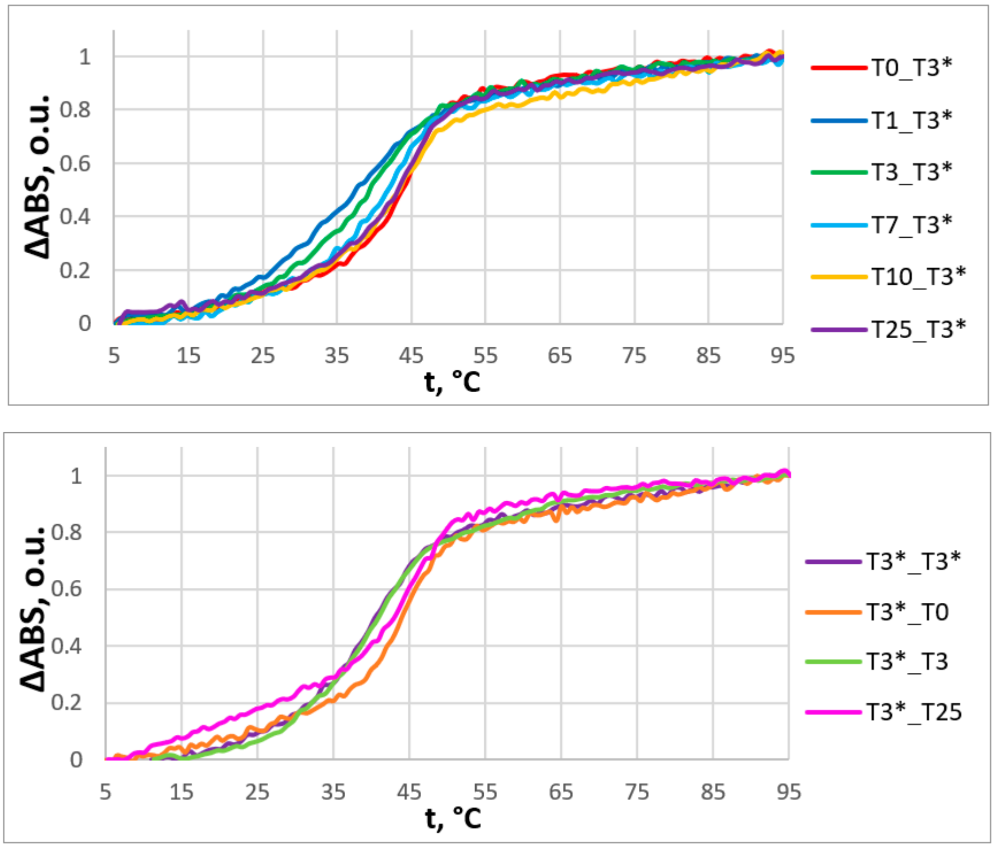

Figure S3. Normalized UV-melting curves for DNA complexes were obtained at 260 nm.

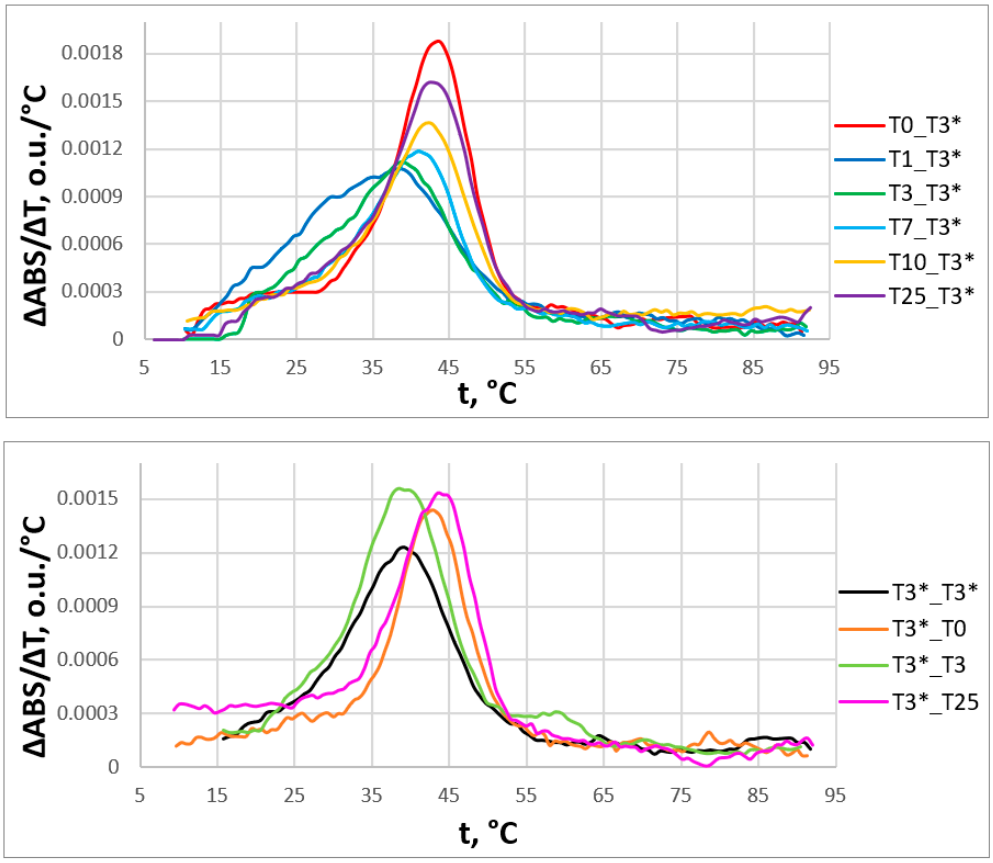

Figure S4. The first derivative of the UV-melting curves, obtained at 260 nm, for DNA complexes.

Data processing of PELDOR data

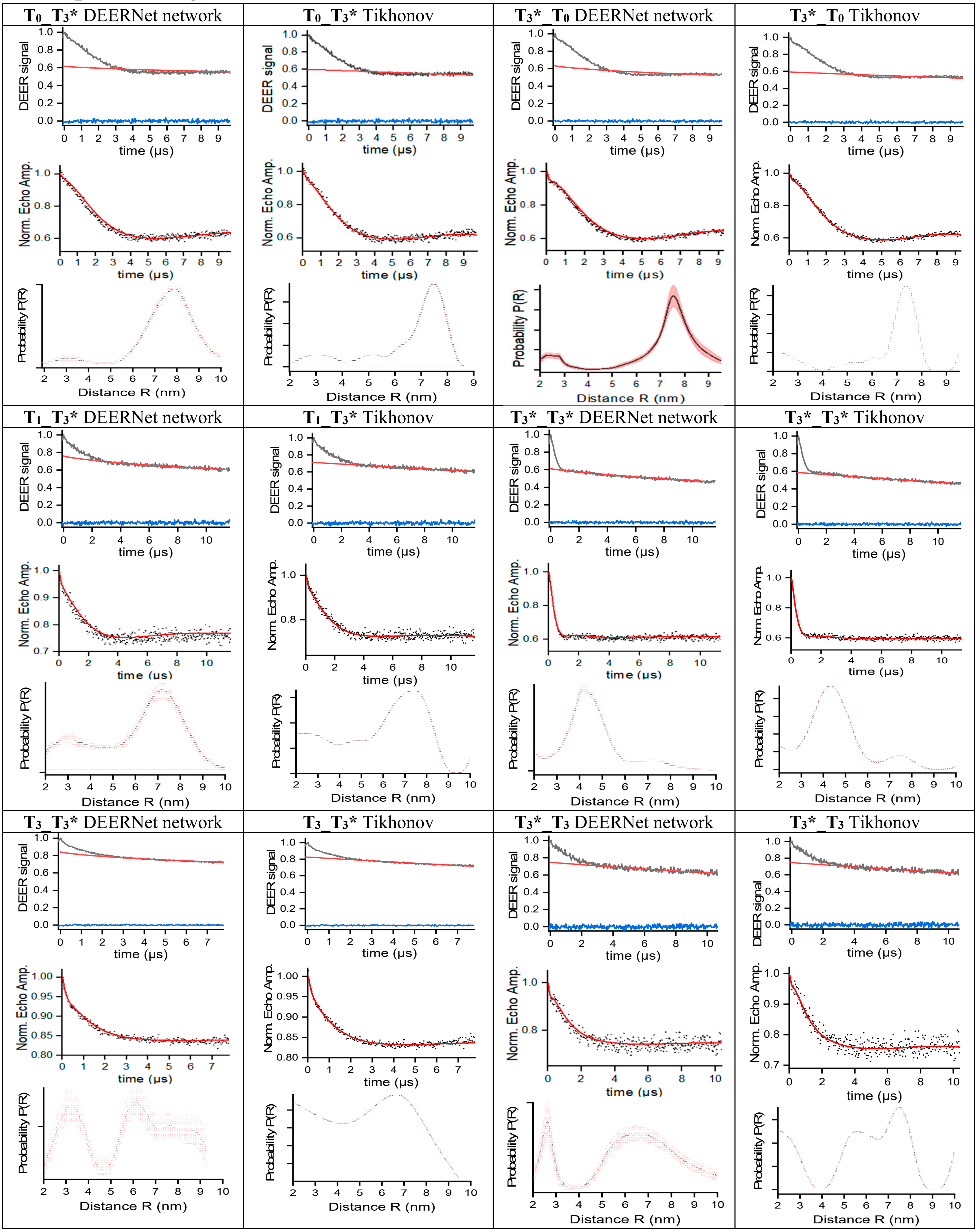

Figure S5. Q-band PELDOR measurements of T_0_T_3*, T_3*_T_0, T_1_T_3*, T_3_T_3*, T_3*_T_3, and T_3*_T_3*. PELDOR time trace (black) with the background fits (red) and an imaginary part of the signal (blue) is shown on the top. Middle background-corrected PELDOR time trace (black dots) and DEERNet or Tikhonov fit (red). The distance distributions on the bottom for each sample are calculated using the DEERNet network or Tikhonov regularization. Shaded areas show the uncertainty of distance determination.

Table S1. Modulation depth extracted by DeerAnalysis software for self-assembled oligonucleotide complexes.

T_0_T_3*	T_1_T_3*	T_3_T_3*	T_3*_T_3*	T_7_T_3*	T_{10}_T_3*	T_{25}_T_3*
0.399	0.288	0.175	0.414	0.092	0.098	0.068
T_3*_T_0		T_3*_T_3				T_3*_T_{25}
0.409		0.254				0.047

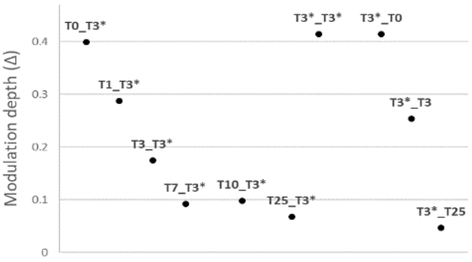

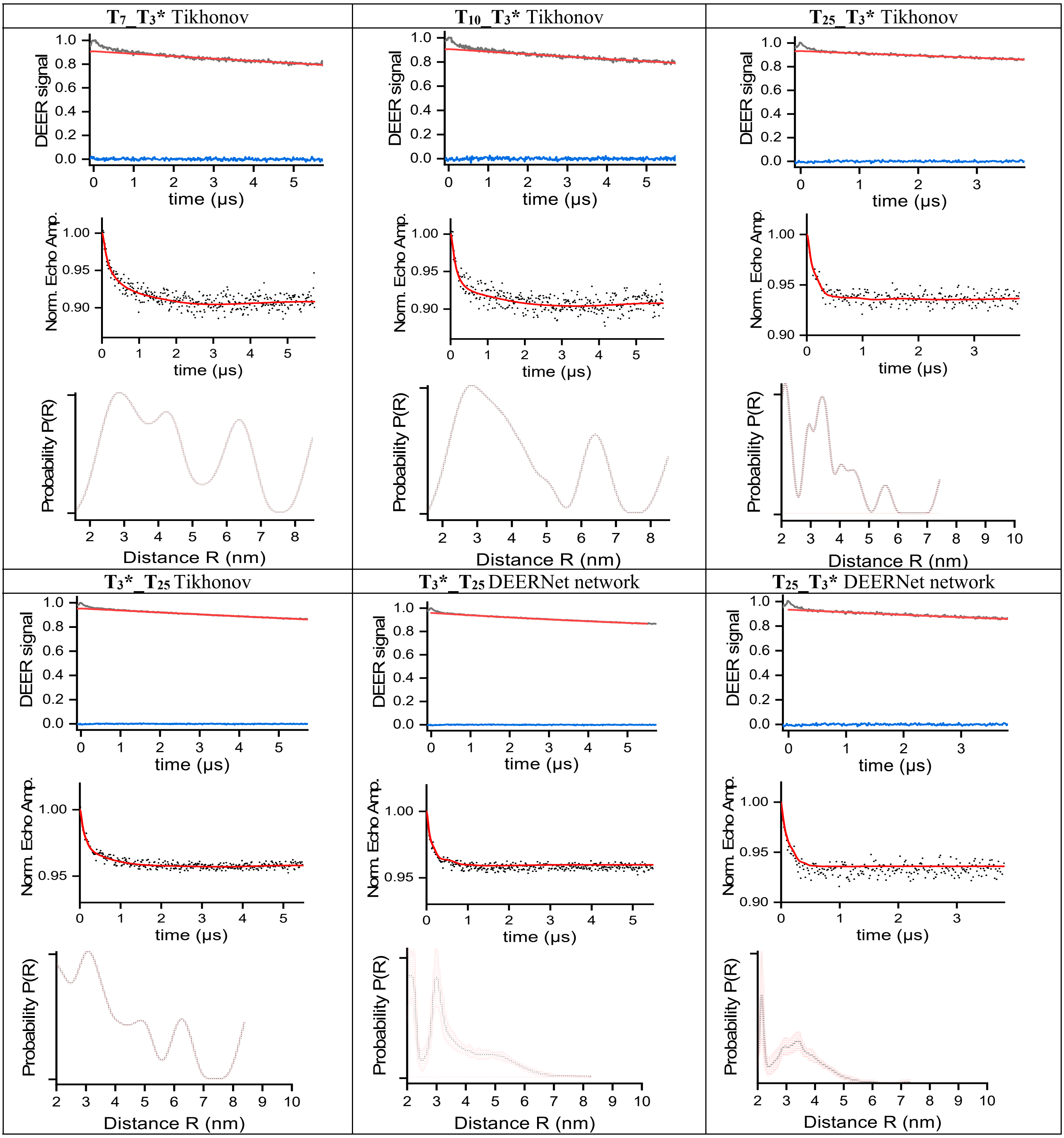

Figure S6. Q-band PELDOR measurements of T_7_T_3*, T_{10}_T_3*, T_3*_T_{25}, and T_{25}_T_3*. PELDOR time trace (black) with the background fits (red) and an imaginary part of the signal (blue) is shown on the top. Middle background-corrected PELDOR time trace (black dots) and DEERNet or Tikhonov fit (red). The distance distributions on the bottom for each sample are calculated using the DEERNet network or Tikhonov regularization. Shaded areas show the uncertainty of distance determination.

Molecular dynamics simulation and analysis

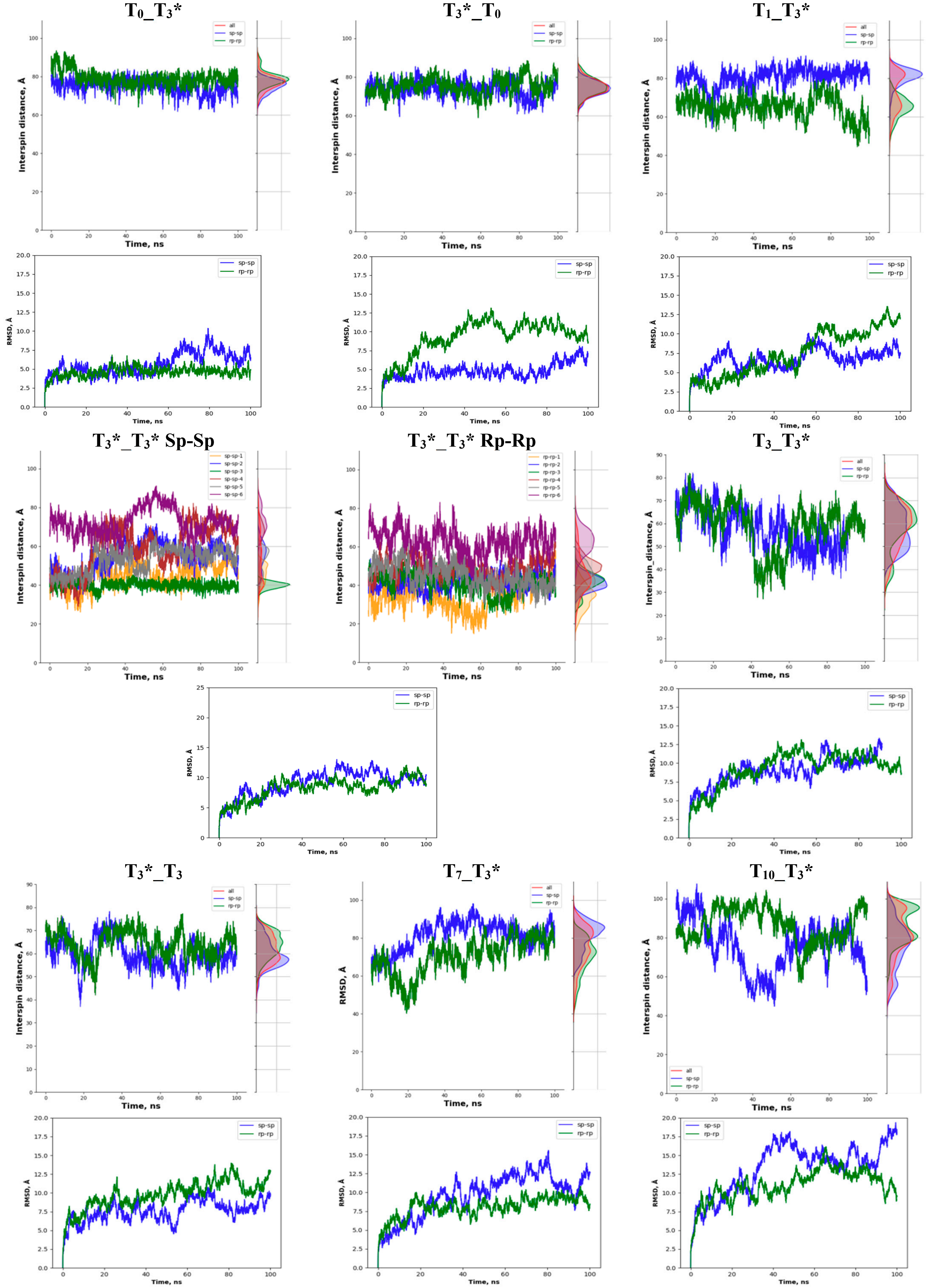

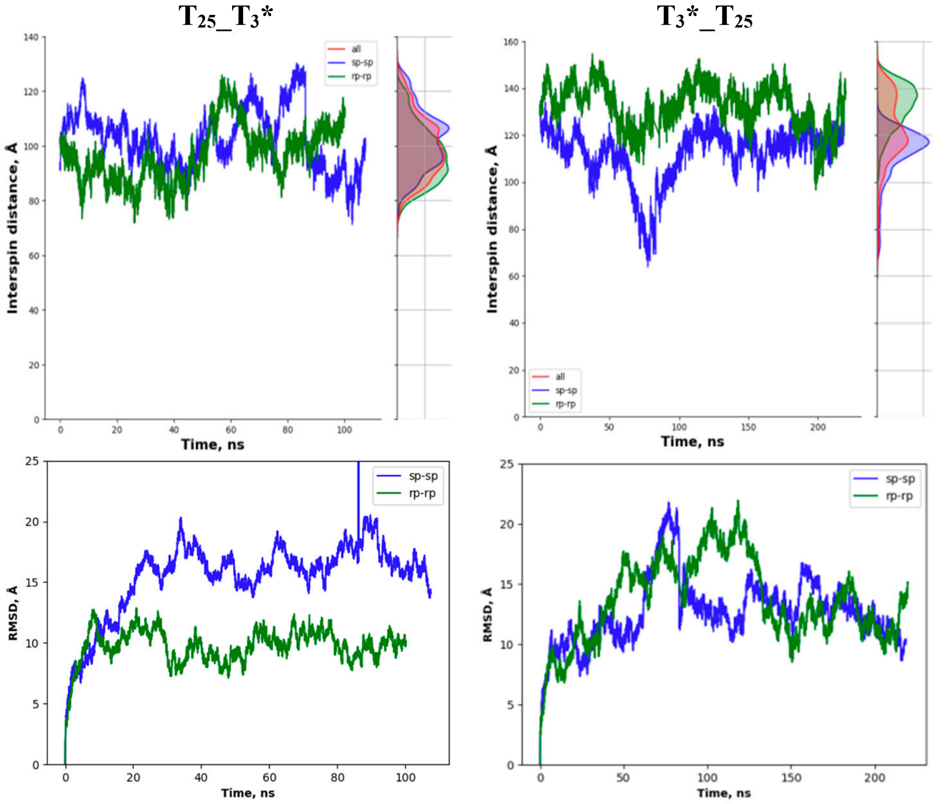

Figure S7. Interspin distances and interspin distance distributions along the molecular dynamics (MD) trajectories for oligonucleotide complexes are on the top. The RMSD values along the MD trajectories are at the bottom for each sample.

$T_0\_T_3*$

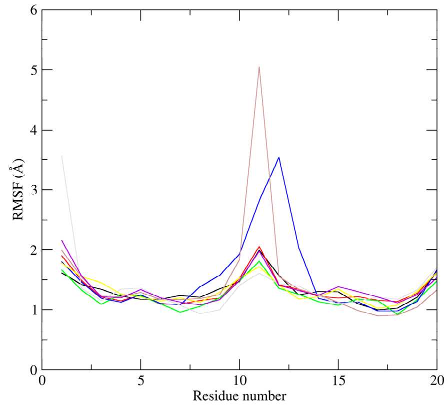

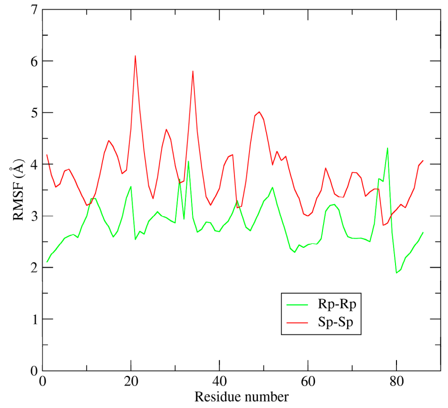

$T_3*\_T_0$

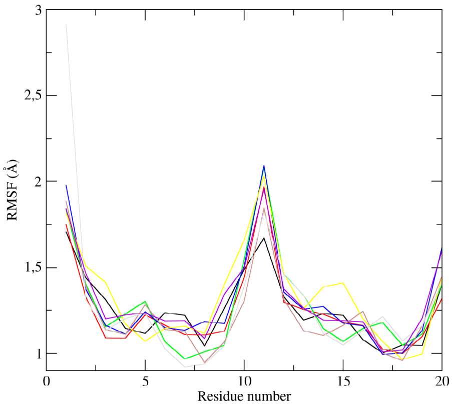

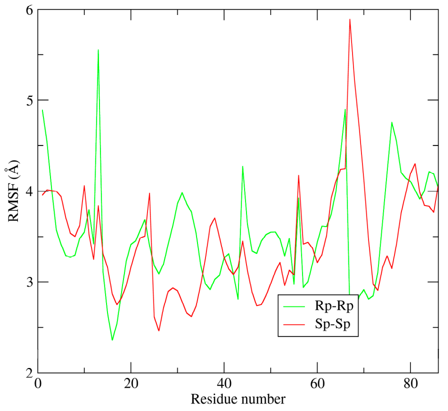

$T_1\_T_3*$

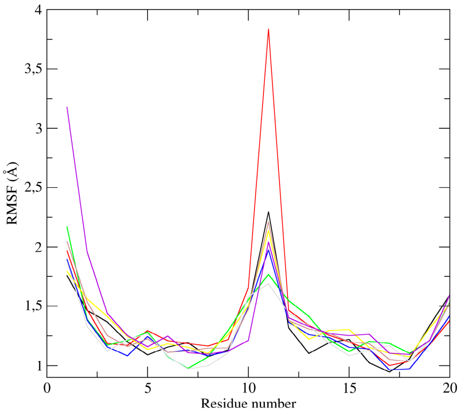

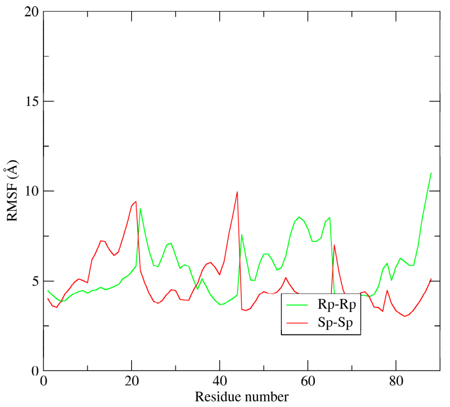

$T_3*\_T_3*$ Sp-Sp

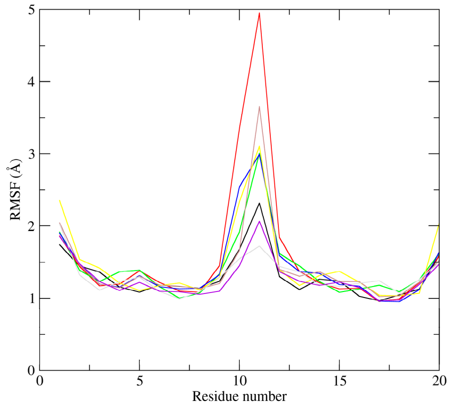

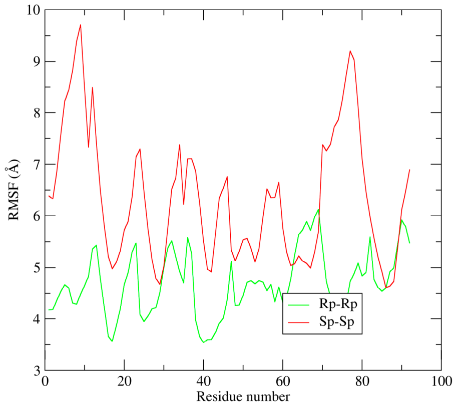

$T_3\_T_3*$

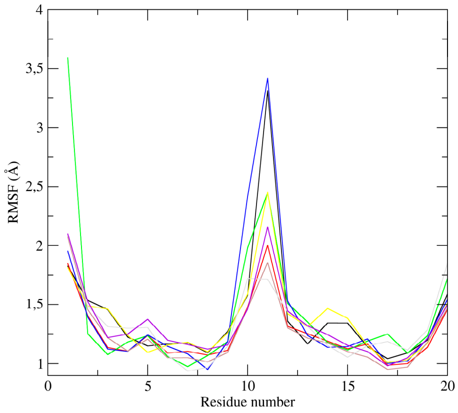

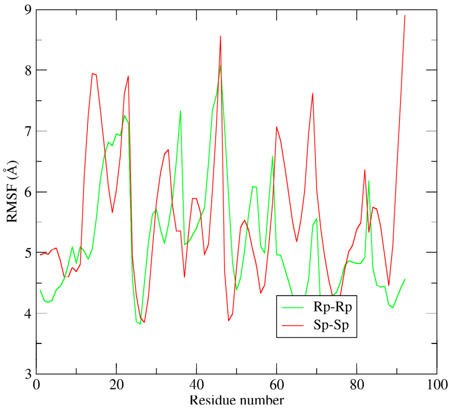

$T_3*\_T_3$

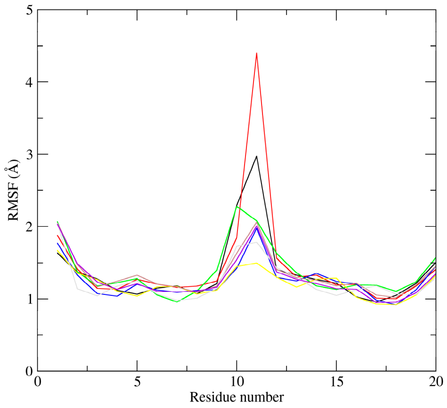

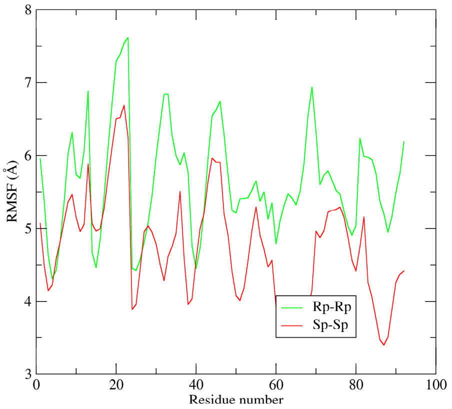

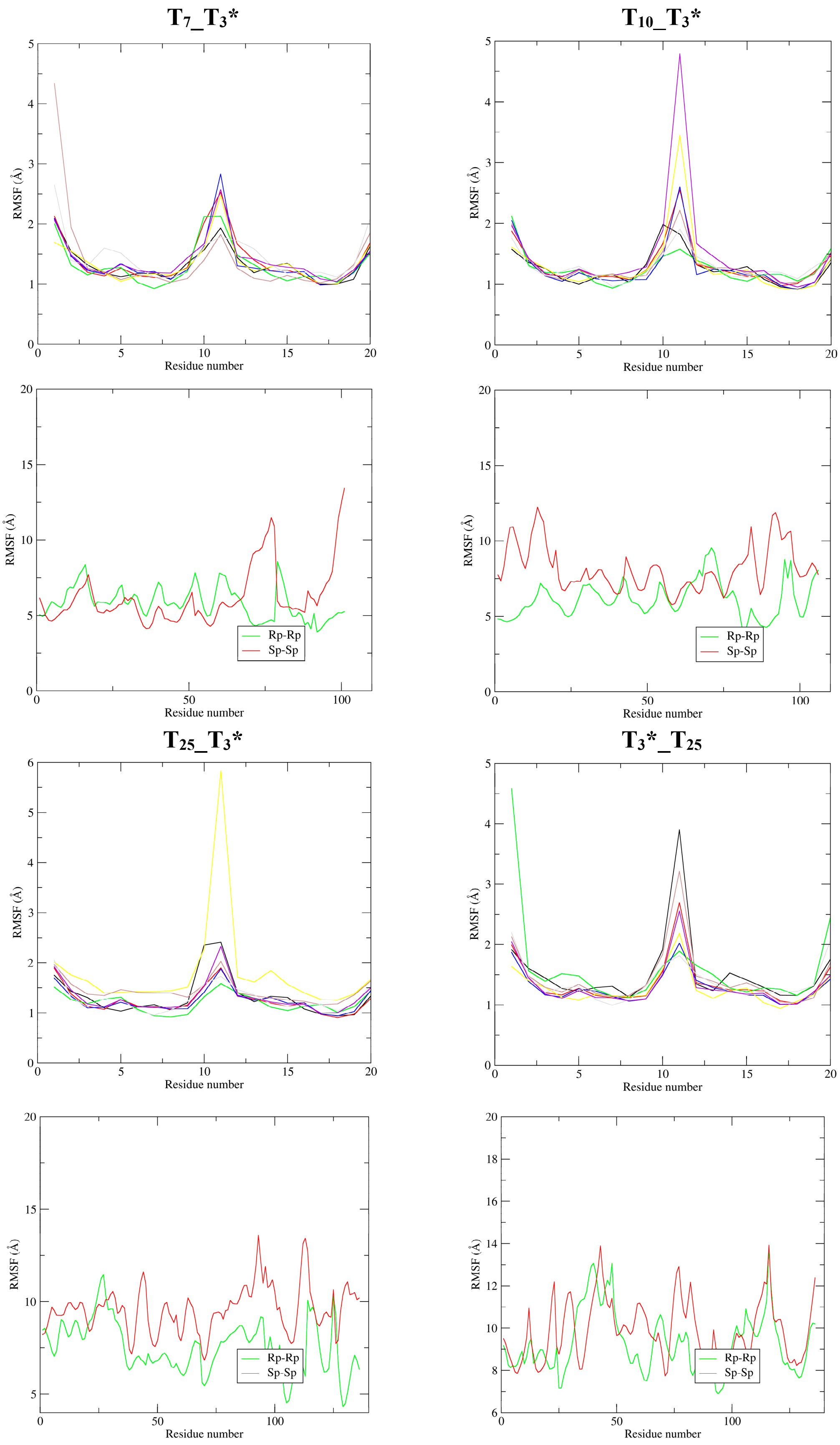

Figure S8. RMSF per residue values for the complex and duplexes in the complex are indicated at the top and bottom for each sample, respectively.

T_0_T_3* Sp-Sp

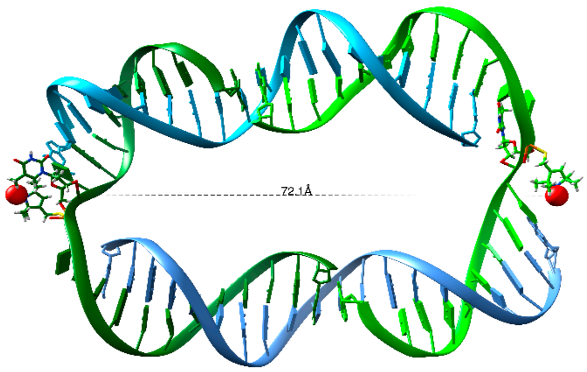

T_0_T_3* Rp-Rp

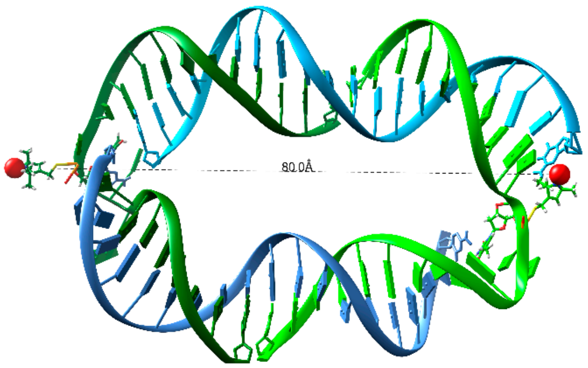

T_3*_T_0 Sp-Sp

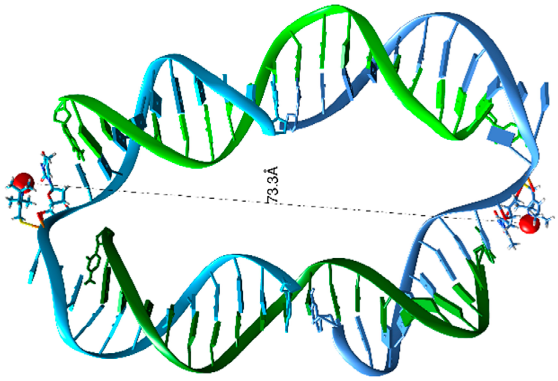

T_3*_T_0 Rp-Rp

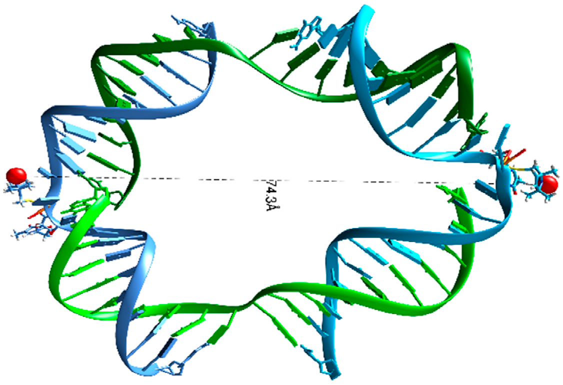

T_1_T_3* Sp-Sp

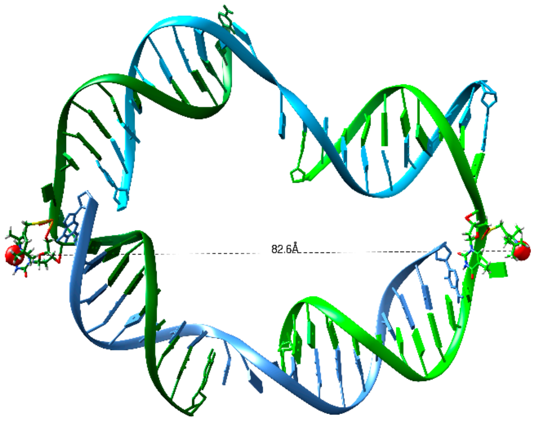

T_1_T_3* Rp-Rp

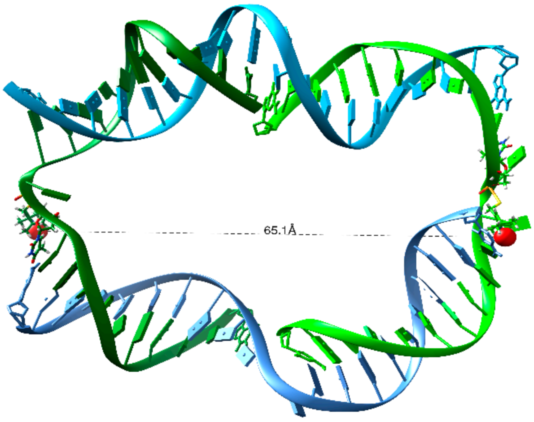

T_3_T_3* Sp-Sp

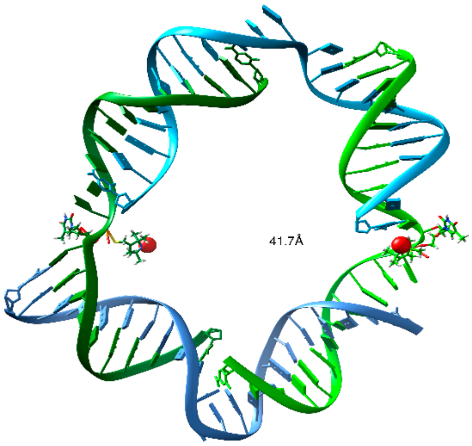

T_3_T_3* Rp-Rp

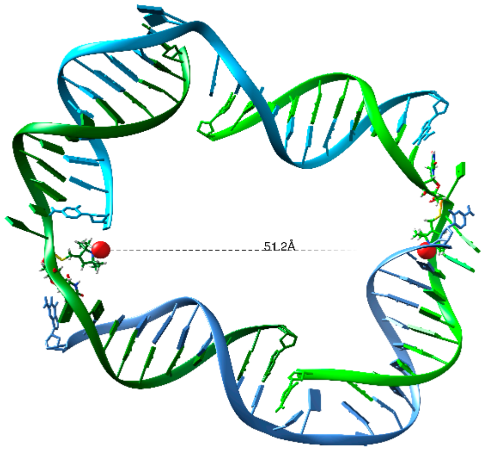

T_3*_T_3 Sp-Sp T_3*_T_3 Rp-Rp

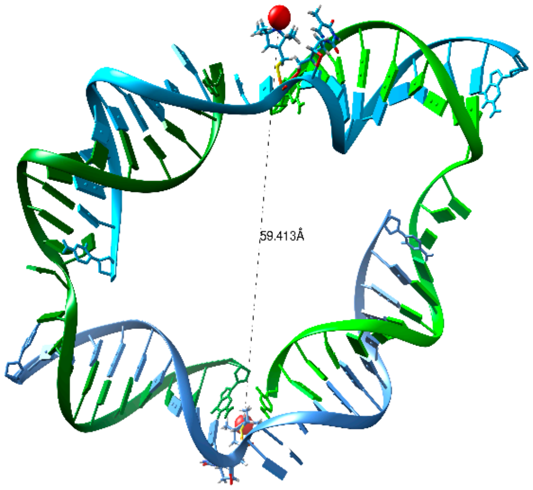

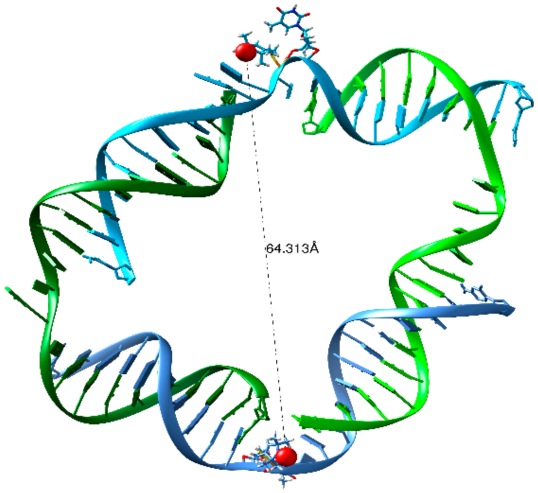

T_3*_T_3* Sp-Sp T_3*_T_3* Rp-Rp

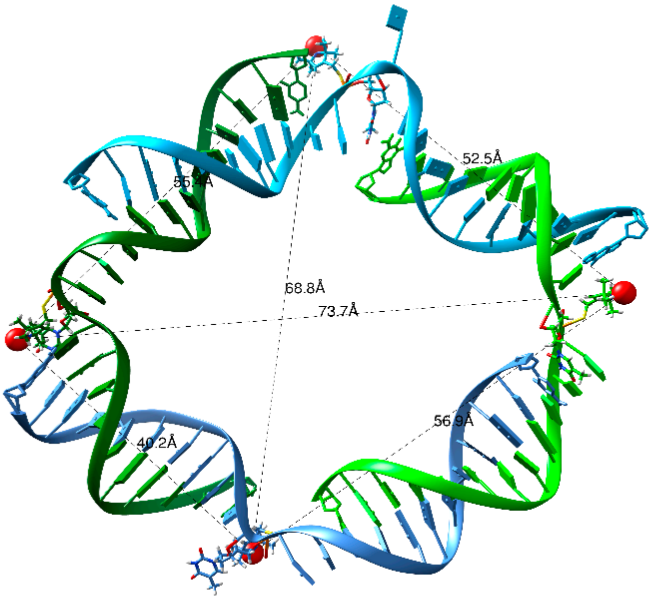

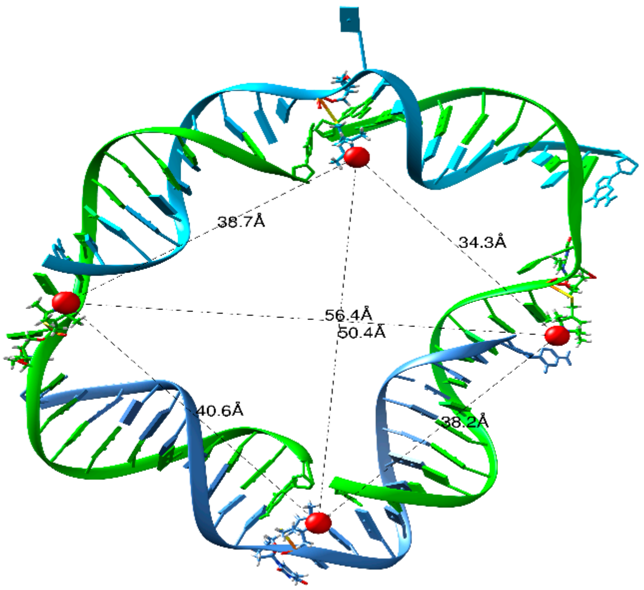

T_7_T_3* Sp-Sp T_7_T_3* Rp-Rp

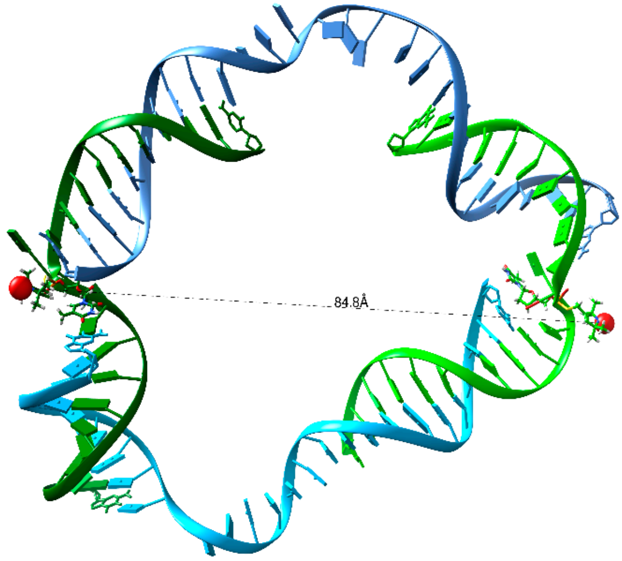

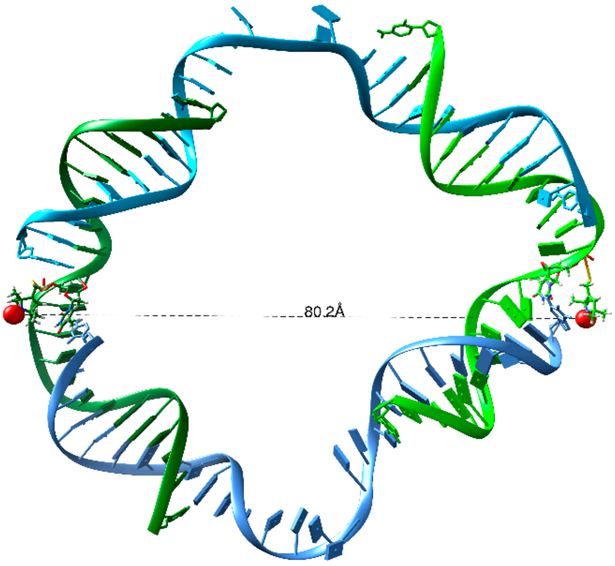

T_{10}_T_3* Sp-Sp T_{10}_T_3* Rp-Rp

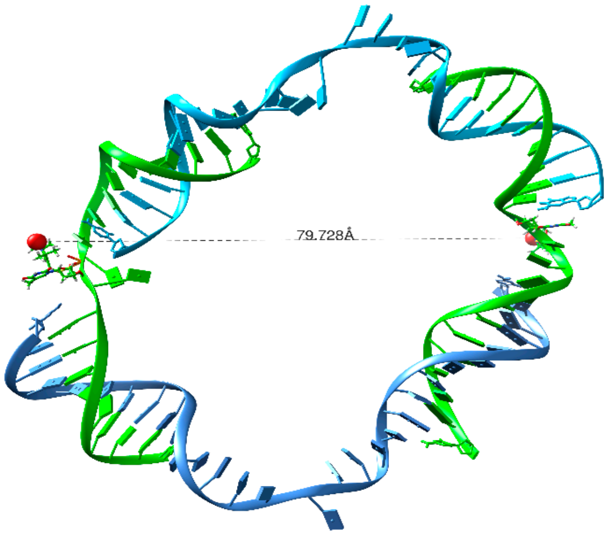

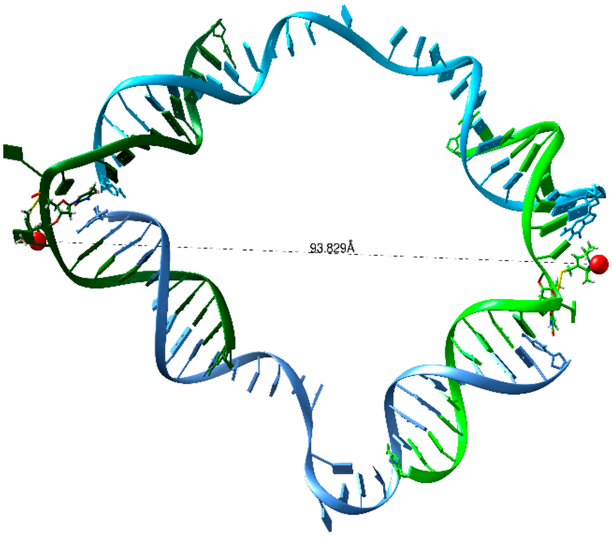

T_{25}_T_3* Sp-Sp **T_{25}_T_3* Rp-Rp**

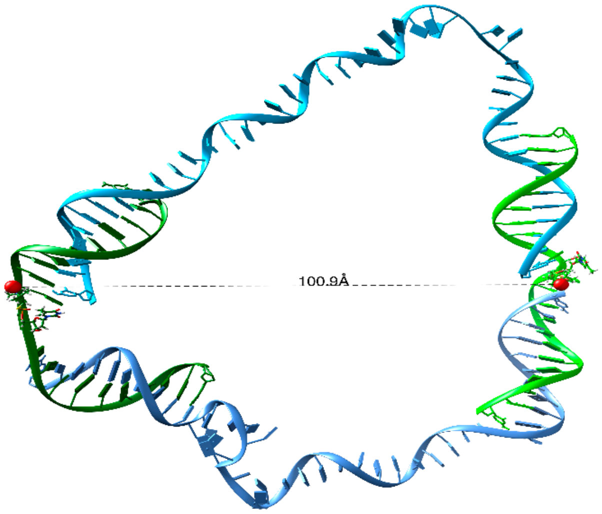

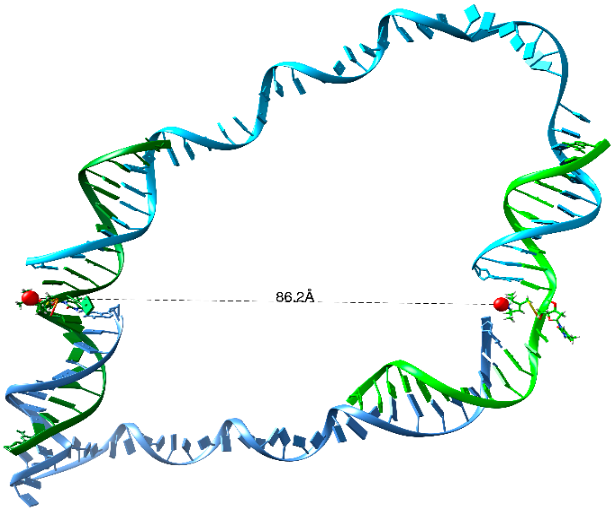

T_3*_T_{25} Sp-Sp **T_3*_T_{25} Rp-Rp**

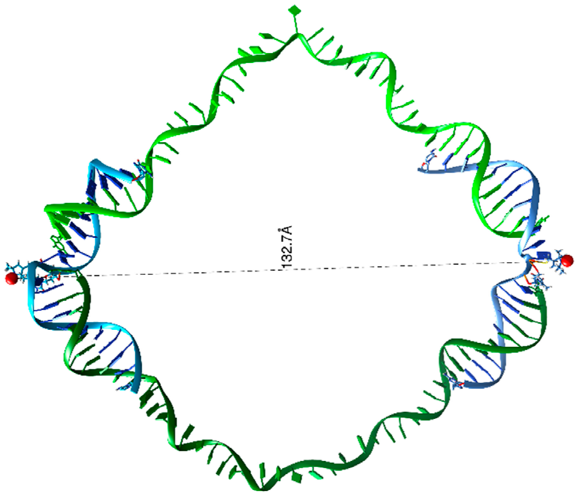

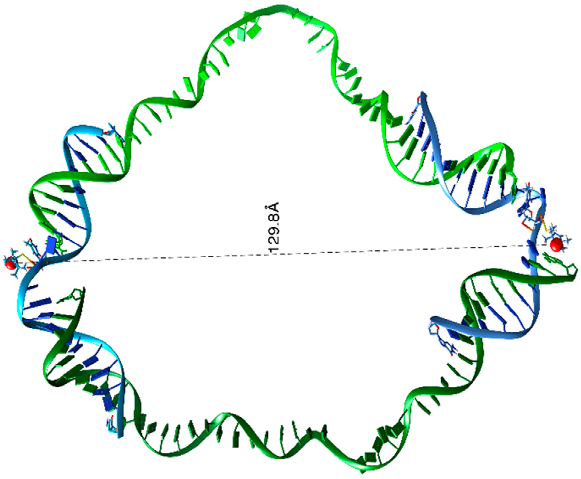

Figure S9. Comparison of the most representative structures in MD trajectories obtained by cluster analysis for oligonucleotide complexes for Sp-Sp and Rp-Rp stereoisomers.

Comparison of PELDOR and molecular dynamics distance distribution for T_7_T_3*, T_{10}_T_3*, T_{25}_T_3*, and T_3*_T_{25} complexes

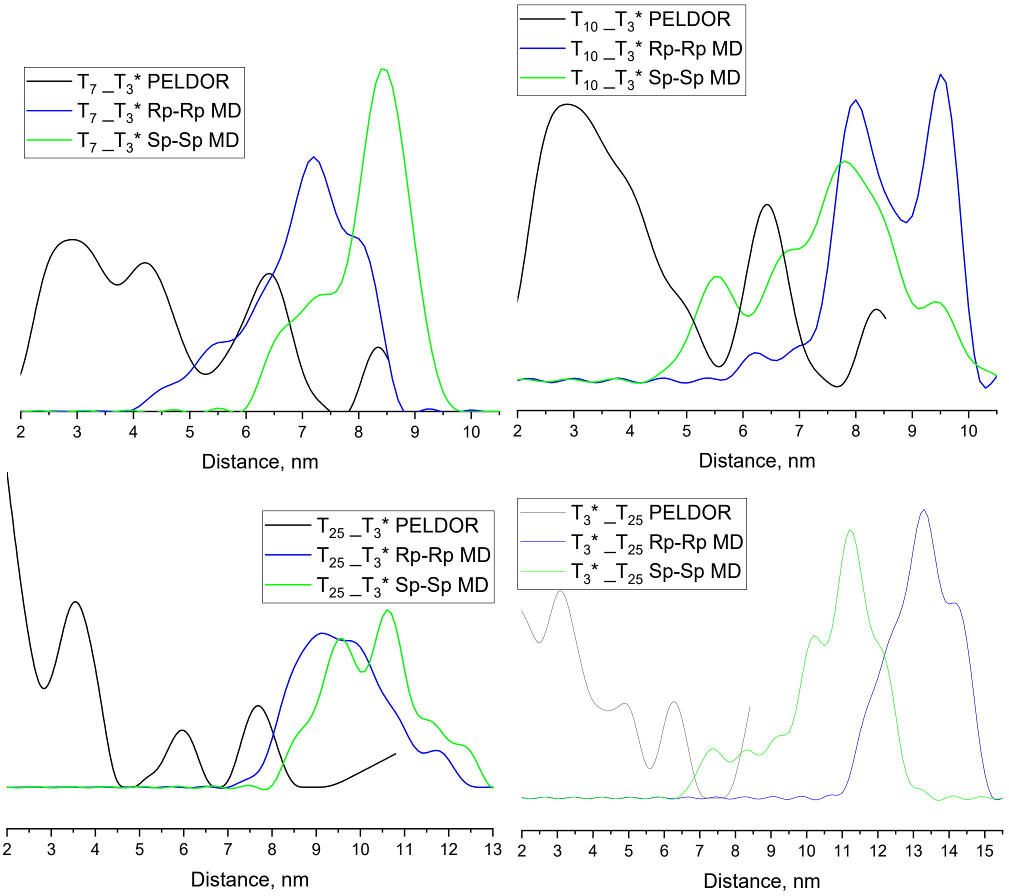

Figure S10. Spin−spin distance distributions for T_7_T_3*, T_{10}_T_3*, T_{25}_T_3*, and T_3*_T_{25} complexes from Tikhonov regularization of PELDOR time traces and MD trajectory analysis.
